# Immobilized metal-affinity chromatography protein-recovery screening is predictive of crystallographic structure success

**DOI:** 10.1107/S1744309111017374

**Published:** 2011-08-13

**Authors:** Ryan Choi, Angela Kelley, David Leibly, Stephen Nakazawa Hewitt, Alberto Napuli, Wesley Van Voorhis

**Affiliations:** a Seattle Structural Genomics Center for Infectious Disease (SSGCID), USA; b Department of Medicine, Division of Allergy and Infectious Diseases, School of Medicine, University of Washington, Box 356423, Seattle, WA 98195-6423, USA

**Keywords:** structural genomics, high throughput, 96-well format, auto-induction, immobilized metal-affinity chromatography, SDS–PAGE, protein expression

## Abstract

The recombinant expression of soluble proteins in *Escherichia coli* continues to be a major bottleneck in structural genomics. The establishment of reliable protocols for the performance of small-scale expression and solubility testing is an essential component of structural genomic pipelines. The SSGCID Protein Production Group at the University of Washington (UW-PPG) has developed a high-throughput screening (HTS) protocol for the measurement of protein recovery from immobilized metal-affinity chromatography (IMAC) which predicts successful purification of hexahistidine-tagged proteins. The protocol is based on manual transfer of samples using multichannel pipettors and 96-well plates and does not depend on the use of robotic platforms. This protocol has been applied to evaluate the expression and solubility of more than 4000 proteins expressed in *E. coli*. The UW-PPG also screens large-scale preparations for recovery from IMAC prior to purification. Analysis of these results show that our low-cost non-automated approach is a reliable method for the HTS demands typical of large structural genomic projects. This paper provides a detailed description of these protocols and statistical analysis of the SSGCID screening results. The results demonstrate that screening for proteins that yield high recovery after IMAC, both after small-scale and large-scale expression, improves the selection of proteins that can be successfully purified and will yield a crystal structure.

## Abstract 2

An overview of the methods used for high-throughput cloning and protein-expression screening of SSGCID hexahistidine recombinant proteins is provided. It is demonstrated that screening for recombinant proteins that are highly recoverable from immobilized metal-affinity chromatography improves the likelihood that a protein will produce a structure.

## 1. Introduction

The greatest challenge, and indeed the most vital requisite, for any laboratory or group involved in structural genomics is the ability to produce hundreds of proteins in parallel (high throughput) and test them using a method that is both cost-effective and reliable in predicting those proteins that can be purified and will yield protein structures (Benita *et al.*, 2006 ▶). Because every protein is structurally unique and characteristically distinct, it is often very difficult to achieve desirable success rates in standardized high-throughput protein-production pipelines, and this is a major contributor to the fiscal and technical burdens faced by many structural genomic projects. Thus, there is a growing demand for the establishment of conditions and methods for expression and screening that (i) are concordant with and can be applied to a great range of proteins and species, (ii) reduce the overall effort and cost of expression trials (Folkers *et al.*, 2004 ▶; Alzari *et al.*, 2006 ▶) and (iii) are a reliable predictor of protein purifications that provide the adequate amounts and quality of protein needed for subsequent successful structural studies (Berrow *et al.*, 2006 ▶).

**Figure 1:**
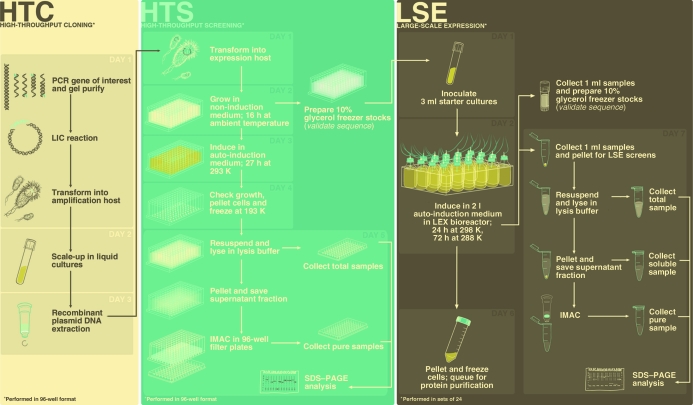
Standard workflow based on UW-PPG’s current cloning, HTS and LSE protocols. Cloning and HTS procedures are carried out manually in 96-well plates and can be completed in two weeks. LSE and screens are performed in sets of 24 using the LEX bioreactor and can be carried out in one week, with the induction step proceeding over the weekend. See Bryan *et al.* (2011 ▶) for a detailed protein-purification workflow.

In an attempt to address these demands, the University of Washington Protein Production Group (UW-PPG), as part of the NIAID-funded Seattle Structural Genomics Center for Infectious Diseases (SSGCID), has designed a non-automated approach to high-throughput screening (HTS) that employs auto-induction methods for the controlled expression of recombinant proteins in *Escherichia coli* (Studier, 2005 ▶), immobilized metal-affinity chromatography (IMAC) for the purification of hexahistidine-tagged proteins and SDS–PAGE analyses for the visual evaluation of expression and recovery levels after IMAC, all of which are performed in a 96-­well format (for a complete workflow, see Fig. 1 ▶). This approach proves to be low-cost and accessible because it does not require the use of expensive robotic platforms. It also allows the entire HTS process, from the transformation of recombinants into host expression strains to the visualization of expression results on protein gels, to be completed in a week, providing reliable predictions of protein-expression and IMAC-recoverability levels for large-scale applications within a reasonable timeframe.

## 2. Materials and methods

### 2.1. High-throughput cloning

#### 2.1.1. Overview of AVA0421 vector features

**Figure 2:**
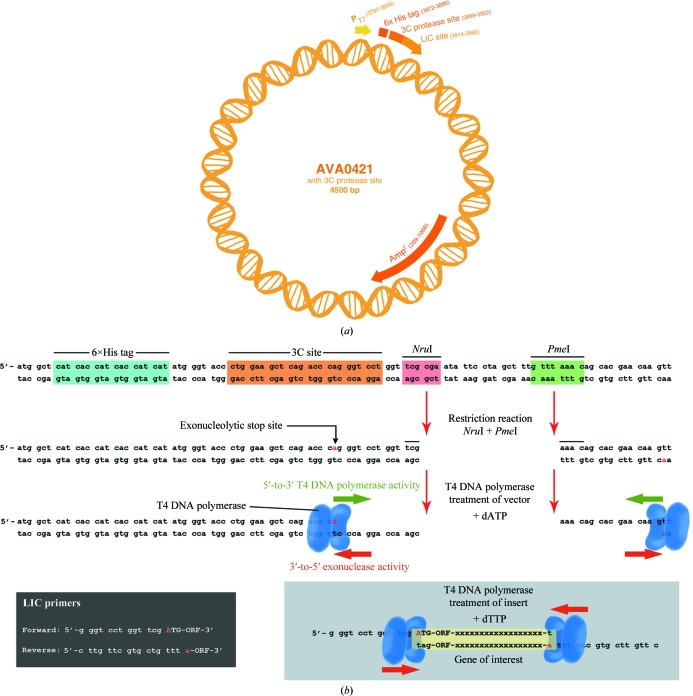
(*a*) AVA0421 vector map. (*b*) Ligation-independent cloning (LIC) site of AVA0421 and LIC-ready reaction of inserts.

Derived from the pET14b vector, the leader sequence of AVA0421 contains a T7 promoter followed by an N-terminal hexahistidine (6×His) nickel-affinity tag and a modified human rhinovirus 3C (HRV-3C) protease recognition site, as well as two restriction sites used for ligation-independent cloning (LIC; Aslanidis & de Jong, 1990 ▶; Alexandrov *et al.*, 2004 ▶; Mehlin *et al.*, 2006 ▶; Quartley *et al.*, 2009 ▶; Fig. 2 ▶). Placement of the 3C cleavage site between the 6×His tag and the open reading frame (ORF) allows the use of subtractive IMAC methods during protein purification to further purify the recombinant protein and remove the cleaved tag (Bryan *et al.*, 2011 ▶). The AVA0421 vector contains the *ampR* gene (also known as *bla*_TEM1_), which confers resistance to ampicillin and carbenicillin for the selection of recombinant constructs during the cloning and expression stages (Fig. 2 ▶ *a*).

#### 2.1.2. Preparation of LIC-ready vector

AVA0421 plasmid DNA was purified from large *E. coli* cultures using a Maxi-Prep kit (Qiagen, Valencia, California, USA). The purified DNA was digested with *Pme*I enzyme (NEB, Ipswich, Massachusetts, USA) and run on a 1% TAE agarose gel (40 m*M* Tris base, 2 m*M* EDTA, 5.7% acetic acid, 1% agarose pH 7) containing ethidium bromide (EtBr; 40 µg l^−1^) for 1.25 h at 150 V. The band of linearized plasmid was excised and gel-purified using a gel-extraction kit (Qiagen, Valencia, California, USA). The purified linear DNA was then digested with *Nru*I enzyme (NEB, Ipswich, Massachusetts) and further purified by ethanol precipitation. Following ethanol precipitation, the DNA concentration was checked using a Nanodrop ND-1000 spectrophotometer (Thermo Scientific, Waltham, Massachusetts, USA) and the DNA was diluted in 1× TE buffer (10 m*M* Tris–HCl, 0.1 m*M* EDTA pH 8) to a concentration of approximately 100 ng µl^−1^. The T4 DNA polymerase-treatment step that followed made use of the exonuclease function of the enzyme to create overhangs on the 5′ vector ends (Fig. 2 ▶ *b*). The reaction was carried out at 295 K for 30 min in the presence of only dATP so that the reaction stopped when the enzyme encountered an adenine nucleotide. The T4 DNA polymerase was heat-inactivated at 348 K for 25 min and 100 µl aliquots of the LIC-ready vector were frozen and stored at 193 K. The LIC-ready AVA0421 remained stable for many months at this temperature and the small size of the aliquots ensured that the LIC-ready vector was generally only thawed once prior to use.

#### 2.1.3. High-throughput cloning procedure

Genes encoding the selected protein targets were PCR-amplified in a 96-well format using either genomic DNA or cDNA as a template, depending on whether introns were predicted to be present. Cycling conditions were chosen based on the GC content of the template(s) being used and an effort was made to group targets with the same template DNA or similar GC content together so that the PCR reactions could be carried out as efficiently as possible (Supplementary Table 1^1^). For targets with a high GC content (>60% of the coding DNA) Phusion polymerase (Finnzymes, Woburn, Massachusetts, USA) and 4% dimethyl sulfoxide (DMSO) was used. Otherwise, Hi-Fidelity polymerase (Roche, Basel, Switzerland) was used without DMSO. The primers used to amplify the insert genes had an LIC sequence appended to their 5′ ends that was complementary to the restriction sites/LIC sequences in the vector. After PCR amplification, the entire 50 µl PCR reaction was run for 1.25 h at 150 V on a 1% TAE agarose gel with EtBr. The PCR products were excised from the gel (after imaging and size verification) and purified using a 96-well gel-extraction kit (Qiagen, Valencia, California, USA). Following this, the products were treated with T4 DNA polymerase (NEB, Ipswich, Massachusetts, USA) in the presence of 2.5 m*M* dTTP to create unique single-stranded overhangs on the 5′ ends of the insert that can pair with the corresponding LIC sites on the digested and T4-treated vector as shown in Fig. 2 ▶. At this point, 2 µl of the T4-treated insert and 1 µl of treated vector were incubated together at ambient temperature, generally 293–295 K, for 5–30 min. The annealing reaction was stopped by the addition of 1 µl 25 m*M* EDTA followed by a heat-shock transformation (10 min on ice, followed by 45 s at 315 K and then ice for 30 min) into NovaBlue *E. coli* amplification strain (EMD Biosciences, Gibbstown, New Jersey, USA). The LIC plasmid constructs were purified from the amplification host using a 96-well Turbo Miniprep kit (Qiagen, Valencia, California, USA). The purified plasmids were then transformed into the expression host strain for expression screening.

### 2.2. High-throughput screening (HTS)

#### 2.2.1. Transformation into expression host strain

The expression of SSGCID proteins requires transformation of clone plasmid DNA into an *E. coli* host that carries the DE3 gene encoding T7 RNA polymerase. SSGCID targets that passed the cloning stage were transformed into BL21(DE3)R3 Rosetta Oxford chemically competent *E. coli* expression strain, which carries the CAT gene that allows chloramphenicol resistance. The cells were prepared in-laboratory, arranged in 96-well plates and stored at 193 K. The transformations were performed by manually transferring 3 µl recombinant plasmid into 120 µl thawed competent cells using an LTS multichannel pipettor (Rainin, Oakland, California) followed by incubation on ice for 20 min and a heat shock at 315 K for 45 s. The cells were left to incubate on ice for a further 20 min. The transformed cells were rescued by pipetting 100 µl into a 96-well deep well block (Costar, Lowell, Massachusetts, USA) containing 500 µl pre-warmed LB medium followed by incubation at 310 K for 1 h on a titer plate shaker (Lab-Line Instruments, Melrose Park, Illinois, USA) with vigorous shaking. The rescued cells were centrifuged briefly at 2000 rev min^−1^ using a Sorvall RC 5C Plus centrifuge fitted with a SH-3000 rotor and a PN11770 96-well plate holder and 500 µl of the supernatant was removed before the cells were resuspended in the remaining medium and plated onto pre-warmed LB–agar with the appropriate antibiotic selection (50 µg ml^−1^ ampicillin, 50 µg ml^−1^ carbenicillin and 34 µg ml^−1^ chloramphenicol; GoldBio, St Louis, Missouri, USA). A positive-control *E. coli* transformant known to express soluble recombinant protein was streaked out from a glycerol stock and screened with the rest of the set to ensure consistency of the HTS. The plates were incubated overnight at 310 K to allow colonies to grow.

#### 2.2.2. Inoculation of non-inducing medium

Using a P20 micro­pipette tip, single colonies from freshly transformed *E. coli* cells were scraped and used to inoculate 820 µl PA-0.5G non-inducing medium [sterile H_2_O, 1 m*M* MgSO_4_, 0.1× metals mix (1000× stock: 50 m*M* FeCl_3_·6H_2_O, 20 m*M* CaCl_2_, 10 m*M* MnCl_2_·4H_2_O, 10 m*M* ZnSO_4_·7H_2_O, 2 m*M* CoCl_2_.6H_2_O, 2 m*M* CuCl_2_·2H_2_O, 2 m*M* NiCl_2_·6H_2_O, 2 m*M* Na_2_MoO_4_·2H_2_O, 2 m*M* Na_2_SeO_3_·5H_2_O, 2 m*M* H_3_BO_3_ in 50 m*M* HCl), 0.5% glucose, 1× NPS (100 m*M* PO_4_^3−^, 25 m*M* SO_4_^2−^, 50 m*M* NH_4_^+^, 100 m*M* Na^+^, 50 m*M* K^+^), 100 mg ml^−1^ l-­methionine and 100 mg ml^−1^ 17 amino-acid mix (1% of each of the following l-amino acids: Na^+^ Glu, Asp, Lys–HCl, Arg–HCl, His–HCl, Ala, Pro, Gly, Thr, Ser, Gln, Asn, Val, Leu, Ile, Phe and Trp)] supplemented with appropriate selection in 96-well blocks. The cultures were grown overnight (about 16 h ) by incubation at ambient temperature, generally 293–295 K, with vigorous mixing using a plate shaker. The next day, the PA-0.5G cultures were incubated for an additional 3 h at 310 K to ensure proper growth of all samples. A 20 µl aliquot of culture was set aside for the inoculation of auto-inducing medium and 10% glycerol stocks were prepared by pipetting 200 µl 80% glycerol and 600 µl PA-0.5G medium into the remaining cultures. The glycerol stocks were flash-frozen in liquid nitrogen and stored at 193 K. DNA-sequence validation of recombinants and subsequent larger scale investigations referred to this glycerol stock for starter cultures.

#### 2.2.3. Inoculation of auto-inducing medium

20 µl of the PA-0.5G cultures was manually transferred into a 96-well block containing 600 µl ZYP-5052 auto-induction medium [Sterile ZY Broth (10 g l^−1^ tryptone, 5 g l^−1^ yeast extract), 1 m*M* MgSO_4_, 1× metals mix, 1× 5052 (0.5% glycerol, 0.05% glucose, 0.2% α-lactose monohydrate) and 1× NPS] supplemented with the correct antibiotics. The block was sealed with an Airpore sheet (Qiagen, Valencia, California) and incubated on a plate shaker inside a refrigerated incubator set at 293 K for roughly 27 h to allow the cultures to reach saturation or early stationary phase. OD_600nm_ values were measured by aliquoting a 1/10 dilution of cells into a flat-bottom 96-well assay plate (Costar, Lowell, Massachusetts, USA) and reading the plates on a Synergy HT multi-mode microplate reader (BioTek, Winooski, Vermont, USA). The cultures were not harvested until OD_600nm_ readings of at least 0.6 were obtained. Once the induced cells were at the correct density, they were centrifuged at 4300 rev min^−1^ for 30 min at 277 K. After centrifugation, the supernatant was discarded and the block with the semi-dry cell pellets was stored at 193 K.

#### 2.2.4. Protein extraction and purification

The cell pellets stored at 193 K were thawed at ambient temperature for 20 min and 600 µl lysis buffer [20 m*M* HEPES pH 7.0, 500 m*M* NaCl, 5% glycerol, 0.5% CHAPS (A.G. Scientific Inc., San Diego, California, USA), 30 m*M* imidazole, 10 m*M* MgCl_2_, 400 µg ml^−1^ lysozyme (Sigma, St Louis, Missouri, USA) and 3 units ml^−1^ Benzonase nuclease (EMD Chemicals, San Diego, California, USA)] was transferred into each well. The cell pellets were then resuspended by pipetting. After resuspension, 600 µl lysis buffer was added to each well and the sample was mixed a second time. The deep well block was then sealed and incubated at room temperature for 1 h on a titer shaker set to moderate. After lysis, a 5 µl sample of the crude lysate was prepared for SDS–PAGE analyses by mixing it with an equal volume of 5× pink reducing sample buffer with DTT (Thermo Scientific, Waltham, Massachusetts, USA) and heating for 5 min at 268 K to denature. The remaining sample was clarified by centrifugation at 4300 rev min^−1^ for 30 min. 825 µl of the soluble supernatant fraction was transferred into a 96-well block pre-loaded with 200 µl pre-equilibrated Ni^2+^ Sepharose beads (GE Healthcare, Piscataway, New Jersey) for HTS IMAC. The protein/resin mixture was incubated for 15 min at 277 K with shaking. The protein/resin mixture was then transferred into a 25 µm 96-well filter plate (Seahorse Labware, North Millerica, Massachusetts, USA). Using a vacuum apparatus, the Ni^2+^ beads were washed three times, each with 1 ml wash buffer (20 m*M* HEPES pH 7.0, 500 m*M* NaCl, 5% glycerol, 30 m*M* imidazole). After washing, IMAC bound proteins were eluted with 100 µl elution buffer, which was identical to the wash buffer with the exception that it contained a higher concentration of imidazole (500 m*M*). 40 µl of the IMAC elution sample was then mixed with 10 µl 2.5× pink reducing sample buffer and denatured for SDS–PAGE analyses.

#### 2.2.5. SDS–PAGE analysis

High-throughput screen (HTS) samples were analyzed by SDS–PAGE using eight Criterion Tris–HCl precast 8–16% gels (pre-run at 100 V for 10 min) with a 26-well comb run on a Criterion Dodeca Cell gel box (Bio-Rad, Hercules, California, USA). Each gel holds the total and IMAC elution fractions of 12 protein targets (or one row of a 96-well plate). The first lane of each gel was loaded with 8 µl Bench Mark Pre-stained Protein Ladder (Invitrogen, Carlsbad, California, USA). Total and IMAC elution fractions for each target were prepared as follows. For the total samples, 5 µl crude lysate was mixed with 5 µl 5× pink reducing sample buffer and was denatured by heating at 368 K for 5 min. 10 µl 1× SDS Tris–glycine running buffer (25 m*M* Tris base, 192 m*M* glycine, 0.1% SDS pH 8.3) was added to dilute the samples and 10 µl of this mixture was loaded onto the gel. For the IMAC-recoverable fractions, 40 µl IMAC elution fraction was mixed with 10 µl 2.5× pink reducing sample buffer and the sample was denatured by heating to 368 K for 5 min. 13 µl of this IMAC elution sample was loaded onto the gel. The gels were run at 200 V for 50 min. After the run was complete, the gels were washed two times for 10 min in H_2_O followed by staining with GelCode Blue protein stain (Thermo Scientific, Waltham, Massachusetts, USA) for 2 h. The gels were destained with H_2_O for 2 h to overnight.

**Figure 3:**
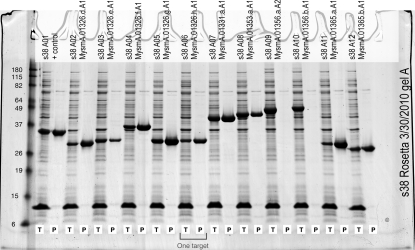
An example of an SDS–PAGE gel displaying the HTS results for 11 *M. smegmatis* target proteins. Reading from left to right, the first lane shows the protein ladder (in kDa). The next two lanes show the total (T) and IMAC elution (P) fractions of a positive control that is known to have high IMAC recovery. The following lanes represent a total of 11 target proteins of two lanes each, alternating between their T and P fractions. IMAC-recovery scores are determined by evaluating the size of each band (as described in §[Sec sec2.2.5]2.2.5), *e.g.* target s38 A03’s recovery would be scored as low, s38 A06’s recovery would be scored as medium and s38 A05’s recovery would be scored as high. Targets s38 A09 and s38 A10 are insoluble and would not be queued for scale up in LSE.

The levels of total and IMAC-recoverable expression detected by SDS–PAGE for each target were scored visually using a standardized criterion (none, no visible bands, no expression or insoluble; low, weak band signifying low expression; medium, adequately sized band, medium expression; high, very large band, high expression; Fig. 3 ▶). The purpose of this system of scoring is to identify those targets that have a sufficient level of soluble expression to be useful for scale up. Generally, target proteins that score low to high for total expression and medium to high for IMAC recoverability are suitable for scaling up. High-priority protein targets that are expressed at medium to high levels but do not appear in IMAC elution fractions and thus are presumed to be insoluble or aggregated are queued for individual­ization of lysis buffers for rescue efforts (Leibly *et al.*, manuscript in preparation).

### 2.3. Large-scale expression (LSE)

#### 2.3.1. LEX bioreactor

**Figure 4:**
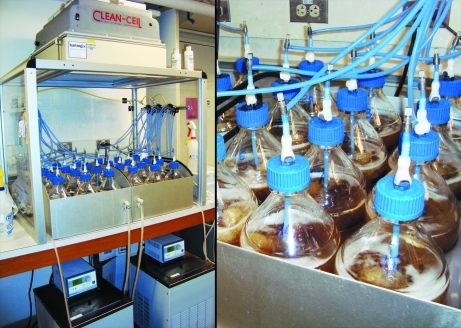
The LEX-48 bioreactor growing 24 individual 2 l cultures. Its overall design features an enclosure with a multi-stage replaceable carbon + HEPA filter forced-air hood, two water circulators, customizable controls for aeration, efficient water-bath regulation of temperature conditions and fully sterilizable components.

In efforts to achieve efficient, reliable and reproducible large-scale expression (LSE) of recombinant proteins, the LEX-48 bench-top bioreactor (Harbinger Biotech, Ontario, Canada) has been utilized for the growth of high-volume bacterial cultures (Fig. 4 ▶). Originally developed by the Structural Genomics Consortium to meet their needs in solving large numbers of protein structures (Vedadi *et al.*, 2007 ▶), the LEX bioreactor efficiently grows up to 48 l of high-density bacterial cultures. Typically, we express 2 l volumes of 24 unique protein targets per LEX run.

#### 2.3.2. Large-scale expression procedure

Starter cultures were prepared by aliquoting 3 ml LB medium containing the appropriate antibiotic selection into 14 ml snap-cap round-bottom tubes (BD Biosciences, San Jose, California, USA) and inoculating from the frozen 10% glycerol stocks prepared during the HTS. The cultures are incubated for 16 h at 310 K with vigorous shaking.

All components of ZYP-5052 auto-induction medium were freshly made every week and prepared for the LEX in the following manner: 1800 ml ZY broth and 200 µl Antifoam 204 (Sigma, St Louis, Missouri, USA) were aliquoted into clean 2 l Pyrex bottles (Corning, Corning, New York, USA) and autoclaved for 90 min to ensure that the medium was fully sterilized; 20× NPS and 50× 5052 stocks were autoclaved for 60 min and all other stocks (1 *M* MgSO_4_, 1000× metals mix and 1000× antibiotics (50 mg ml^−1^ ampicillin, 50 mg ml^−1^ carbenicillin, 34 mg ml^−1^ chloramphenicol) were sterilized through a 0.22 µm filter (Millipore, Carrigtwohill, Ireland). On the day of LEX inoculation, the components were mixed to produce the final medium to be used. The 3 ml starter cultures were added to the bottles, sealed with sparger caps and placed in the LEX bioreactor. The airflow was adjusted evenly for each bottle and they were left to incubate at 298 K for 24 h followed by drop in temperature to 288 K for 72 h. To reduce the chance of contamination, the bottles were not opened until harvest.

OD_600nm_ values were not typically monitored for the assessment of growth for LSEs. Alternatively, at harvest, the LEX cultures were transferred to clean 2 l centrifuge buckets, pelleted at 4000*g* using a Sorvall RC 12 BP centrifuge fitted with an H-12000 swinging-bucket rotor and the masses of the cell pastes were measured to verify proper growth (usually in the range of 20–30 g per 2 l culture). The pelleted cells were flash-frozen in liquid nitrogen and stored at 193 K until they were selected for protein purification.

#### 2.3.3. LSE screens

**Figure 5:**
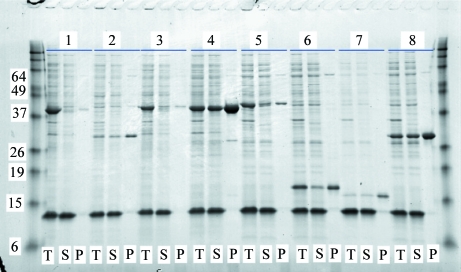
An example of an SDS–PAGE gel of LSE screens of eight expressed *M. smegmatis* target proteins. The two outermost lanes hold the protein ladders (labeled in kDa). Each target protein-expression preparation occupies three lanes: total expressed (T), soluble expressed (S) and IMAC elution pure (P) fractions. The variations in expression levels as seen in this gel are typical. The solubility-scoring system is identical to that of the HTS (as described in §[Sec sec2.2.5]2.2.5). Target protein recoverability after IMAC would be scored as low for 2, 3 and 7, medium for 5 and 6, and high for 4 and 8. Target 1 is primarily insoluble and would not be queued for purification.

All LSEs were screened prior to protein purification in order to verify expression and IMAC-recovery levels as predicted by the HTS. Before harvest, 1 ml aliquots were removed from the 2 l LEX bottles, pelleted at 4000*g* for 20 min and stored at 193 K. The pellets were subsequently thawed at ambient temperature and resuspended and lysed for 1 h in 3 ml lysis buffer. For the preparation of SDS–PAGE analyses, a 4 µl (‘total’) sample was taken from the cell lysate; the remaining sample was centrifuged at 4000*g* for 30 min and a 4 µl (‘soluble’) sample was taken from the supernatant fraction; 700 µl of the remaining supernatant was exposed to 100 µl pre-equilibrated Ni^2+^ Sepharose beads, washed with 2.1 ml wash buffer, eluted with 100 µl elution buffer and a 10 µl IMAC elution (‘pure’) sample was taken. All fractions were mixed with 5× pink reducing sample buffer, denatured by boiling and analyzed *via* SDS–PAGE and scored as described in §[Sec sec2.2.5]2.2.5 (Fig. 5 ▶).

## 3. Results and discussion

During the past three years, the SSGCID protein-production pipeline has conducted thousands of HTSs and LSEs to test the expression and IMAC recoverability of recombinant proteins expressed in *E. coli* in both small-scale and large-scale culture formats. Here, we present analysis of these results to evaluate the success of SSGCID HTS, to better understand the correlation between HTS results and LSEs, and to determine the value of performing screens for IMAC-recoverable proteins in both small-scale and large-scale expression. The first goal of these analyses was to generate an accurate account of the success rate of HTS as defined by the total number of SSGCID clones with associated HTS results. From the commencement of the SSGCID project in February 2008 to December 2010, the UW-PPG high-throughput cloning pipeline has been successful in producing 4627 unique clones; of these, 4330 (94%) were effectively transformed into the BL21(DE3)R3 Rosetta Oxford expression strain and passed on to the HTS pipeline (these figures do not include those protein targets that failed during the initial cloning steps nor any of their subsequent rescue attempts). The 6% failure rate can be attributed to several different factors, *e.g.* poor quality of plasmid DNA, low efficiencies of competent cell stocks, the expression of proteins that are toxic to *E. coli* and/or human error. A chronological review of HTS results suggests that the number of clones that failed at the expression host transformation step has decreased over time. In fact, analysis of the last six months reveals the current failure rate to be under 1%. The improved success rate was likely to be a result of practical experience gained during the first three years of the project and the careful optimization of standard operating procedures (SOP), which ultimately led to the SOP described in this paper. Furthermore, the low failure rate suggests that a manual non-automated approach can be a reliable method for HTS applications.

**Figure 6:**
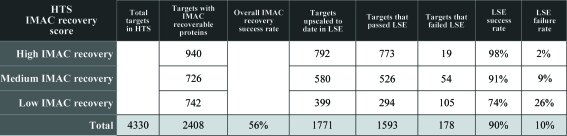
HTS IMAC recovery results (high, medium or low) and LSE screening success rates.

**Figure 7:**
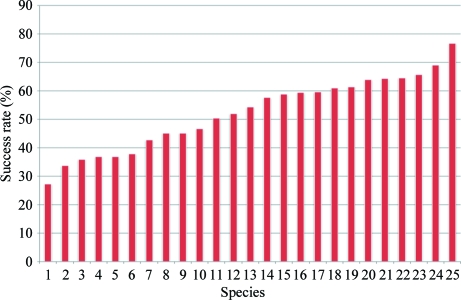
HTS IMAC recovery success rates (*y* axis) of 25 commonly screened species (*x* axis), where HTS success is defined by the number of preparations that had either low, medium or high IMAC recovery divided by the total screened and multiplied by 100. 1, *Plasmodium falciparum*; 2, *Coccidioides immitis*; 3, *Mycobacterium bovis*; 4, *M. leprae*; 5, *Toxoplasma gondii*; 6, *M. ulcerans*; 7, *Borrelia burgdorferi*; 8, *Anaplasma phagocytophilum*; 9, *M. tuberculosis*; 10, *Entamoeba histolytica*; 11, *Rickettsia prowazekii*; 12, *Babesia bovis*; 13, *Encephalitozoon cuniculi*; 14, *M. thermoresistible*; 15, *M. avium*; 16, *Cryptosporidium parvum*; 17, *M. abscessus*; 18, *Burkholderia pseudomallei*; 19, *Bartonella henselae*; 20, *M. marinum*; 21, *Ehrlichia chaffeensis*; 22, *M. paratuberculosis*; 23, *Giardia lamblia*; 24, *Brucella abortus*; 25, *M. smegmatis*.

**Figure 8:**
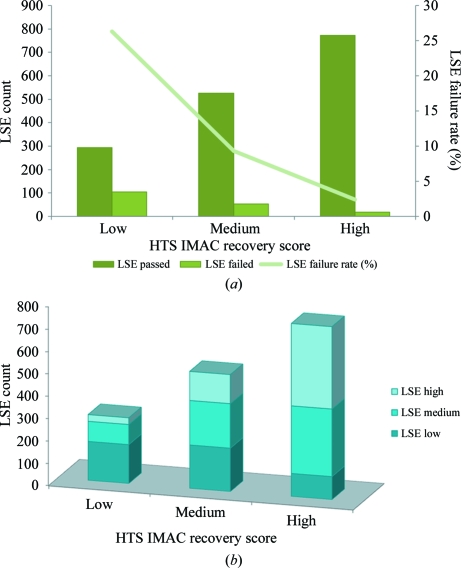
(*a*) Relationship between HTS IMAC-recovery scores (*x* axis) and LSE success (*y* axis, left) and failure rates (*y* axis, right). (*b*) A comparison of HTS IMAC-recovery scores (*x* axis) and LSE IMAC-recovery results (*y* axis).

**Figure 9:**
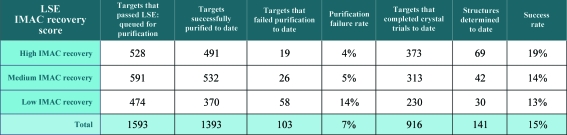
LSE screening IMAC-recovery results, protein-purification and structure-determination success rates.

Upon visual analyses of SDS–PAGE gels of the 4330 clones that passed through the HTS pipeline, approximately 56% were observed to produce protein that could be recovered after elution from IMAC (IMAC-recoverable protein targets). Of these, 39% were scored as high-recovery, 30% as moderate-recovery and 31% as low-recovery protein targets (Fig. 6 ▶). An important measure of the strength of a standardized protocol for high-throughput protein production is its applicability to a variety of species. Our HTS SOP has been successful in predicting IMAC-recoverable protein levels in over 30 different species. Of the 25 most commonly screened species, results show that the HTS IMAC-recoverable protein success rate, where success is considered as high, medium or low recovery, ranges from 27% for *Plasmodium falciparum* to 77% for *Mycobacterium smegmatis* (Fig. 7 ▶). The purpose of HTS is to identify good candidates for LSE based on their recovery from small-scale IMAC. However, a comparison of HTS results with those from LSE revealed that some protein targets that scored as IMAC-recoverable proteins in HTS failed to be IMAC recoverable at the LSE step. To date, we have completed 1771 LSEs for protein targets that have shown IMAC-recoverable proteins in HTS and 178 of these protein targets failed to be recoverable at the LSE step, which represents a failure rate of 10% (Fig. 6 ▶). Further analysis revealed a strong correlation between HTS recovery scores and LSE failures. The majority of the protein targets that failed recovery at the LSE step had low HTS scores (61%), followed by targets with medium (30%) and high (9%) HTS scores (Fig. 6 ▶). Dividing the results of the LSEs by their HTS IMAC-recovery scores, those LSEs with low HTS scores had a 26% failure rate, those LSEs with a medium HTS score had a 9% failure rate and those with a high HTS score had only a 2% failure rate (Fig. 8 ▶ *a*). Furthermore, a comparative assessment of HTS and LSE IMAC-recovery scores demonstrates that HTS successfully predicted high and low recovery levels in LSE in 48% and 59% of the cases, respectively (Fig. 8 ▶ *b*). However, HTS predictions for protein targets with medium scores were not as consistent with LSE screening results. While 38% of these HTS medium-scored protein targets gave comparable medium scores in LSE, 37% were found to have low LSE scores and 25% were found to have high LSE scores. We would not want to exclude these high LSE-scoring proteins that would be lost if we did not upscale medium-scoring HTS proteins, as the high LSE-scoring proteins are 39% more likely to yield crystal structures than medium or low recovery scored LSE proteins (Fig. 9 ▶; see discussion in the next paragraph). This discordance between HTS and LSE screening may be attributed to a number of factors, including differences in growth, sample preparation and handling methods used for HTS and LSE, and perhaps inconsistencies arising from the somewhat subjective nature of the scoring method. Nevertheless, these results indicate that HTS screening was valuable in predicting which clones were worthwhile in performing LSEs. The results suggest that if LSEs are limiting and all other factors are equal, we should favour medium and high HTS scores for LSEs to avoid high failure rates and low expression while capturing the maximum number of high IMAC-recoverable proteins in LSE, as these are the most likely to yield protein structures.

Comparisons between LSE scores and successful protein purifications reveal that failure rates for purification are higher for targets with low LSE scores (14%) than protein targets with medium (5%) and high scores (4%) (Fig. 9 ▶). Thus, selecting proteins with medium and high LSE scores would reduce the failure rate of purification by about two-thirds compared with the failure rate of low expressors. Analysis of LSEs that led to protein structures demonstrates that proteins with high scores in LSEs had a 19% probability of producing a structure, compared with proteins with low and medium LSE scores which had a 13–14% probability of yielding a structure (Fig. 9 ▶). Thus, a protein with a high soluble LSE score was approximately 39% more likely to give a structure than a protein with a medium or low soluble LSE expression score. Since one goal of our structural genomics group is to produce as many structures as possible, the results suggest that high IMAC-recoverable proteins in LSE screens should be prioritized for purification and crystal trials if all other priorities are equal. In conclusion, we feel that the results presented demonstrate value for both the HTS and LSE protein-screening assays and we plan to continue both screens. To achieve maximal success and efficiency, all protein targets that show high or medium recovery scores in HTS will be prioritized for LSE and high IMAC-recoverable proteins in the LSE screen will be prioritized for purification and crystallography trials.

## Supplementary Material

Supplementary material file. DOI: 10.1107/S1744309111017374/en5455sup1.pdf

## Footnotes

1 Supplementary material has been deposited in the IUCr electronic archive (Reference: EN5455).
