# Supplementary material for: Immobilized metal-affinity chromatography protein-recovery screening is predictive of crystallographic structure success
Source: Acta Crystallogr Sect F Struct Biol Cryst Commun. 2011 Aug 13;67(Pt 9):998–1005. doi: 10.1107/S1744309111017374 (PMC3169392; doi:10.1107/S1744309111017374)
Supplement: Supplementary file 1 [file f-67-00998-sup1.pdf]

High-throughput Cloning: PCR Cycling Conditions

	Standard	A-T Rich	G-C Rich
	Roche<br>Hi-Fidelity polymerase	Roche<br>Hi-Fidelity polymerase	Finnzymes<br>Phusion polymerase<br>w/ 4% DMSO
1)	94°C for 2:00 min	94°C for 2:00 min	98˚C for 0:30 sec
2)	94°C for 0:30 sec	94°C for 0:20 sec	98˚C for 0:15 sec
3)	60°C for 1:00 min	2.1° per sec to 50°C	65˚C for 0:15 sec
4)	72°C for 4:30 min	50°C for 0:10 sec	72˚C for 0:30 sec
5)	Go to 2, 29 times	2.1° per sec to 37°C	Go to 2, 29 times
6)	72°C for 10:00 min	37°C for 0:10 sec	72˚C for 3:00 min
7)	4°C for ever	2.1°per sec to 60°C	4˚C for ever
8)	End	60°C for 5:00 min	End
9)		Go to 2, 4 times	
10)		94°C for 0:20 sec	
11)		2.1° per sec to 50°C	
12)		50°C for 0:10 sec	
13)		2.1°per sec to 42°C	
14)		42°C for 0:10 sec	
15)		2.1° per sec to 60°C	
16)		60°C for 5:00 min	
17)		Go to 10, 28 times	
18)		60°C for 10:00 min	
19)		4°C for ever	
20)		End	
